# The “Lasso-Technique”: A Maneuver to Stabilize a Steerable Sheath for Transfemoral Access to Antegrade Branches in Branched Endovascular Aortic Repair—A Report of Four Cases

**DOI:** 10.1177/15266028251334265

**Published:** 2025-09-24

**Authors:** Heiko Wendorff, Maryna Jensch, Felix Kirchhoff, Michael Kallmayer, Daniela Branzan, Angelos Karlas, Christoph Knappich

**Affiliations:** 1 Clinic and Polyclinic for Vascular and Endovascular Surgery, TUM University Hospital, Hospital Rechts der Isar, Technical University of Munich, Munich, Germany; 2 Chair for Computer Aided Medical Procedures & Augmented Reality, Technical University of Munich, Munich, Germany; 3 DZHK (German Centre for Cardiovascular Research), partner site Munich Heart Alliance, Munich, Germany

**Keywords:** Snare, bailout technique, catheterization, thoracoabdominal aortic aneurysm, aneurysm repair, branched stent graft, angiography, endovascular therapy, EVAR

## Abstract

**Purpose:**

To describe and share our experience of the “Lasso-Technique,” employing a Snare® catheter combined with a small-sized 7F steerable sheath to facilitate transfemoral access to antegrade aortic branches during branched endovascular aortic repair.

**Background:**

Cannulation of antegrade branches of a thoracoabdominal aortic stent graft via transfemoral access is technically challenging, even with a steerable sheath. For example, introducing a bridging stent may deflect the sheath cranially and prohibit successful implantation. To avoid alternative brachial access and associated complications, we employed and presented herein the “Lasso-Technique.”

**Technique:**

The tip of the 7F steerable sheath is caught using the Snare® catheter (introduced via a contralateral femoral access with a minimum sheath size of 4F), which is then locked by a mosquito clamp. While introducing the bridging stent into the target branch, the assistant stabilizes the sheath by slightly pulling the Snare-catheter.

**Conclusion:**

The presented approach is an easily accomplished maneuver enabling transfemoral implantation of bridging stents in thoracoabdominal aortic stent grafts. Compared to similar techniques, it requires sheaths with smaller diameters (7F and 4F vs 12F) yet bilateral femoral artery puncture. It allows for hooking the sheath in the target branch, offering increased stability while being safer compared to upper extremity access approaches.

**Clinical Impact:**

The “Lasso-Technique” is a practical and reproducible method for accessing branches during branched endovascular aortic repair (b-EVAR) procedures. The technique eliminates the need for brachial access, potentially reducing radiation exposure and complications associated with upper-extremity access. It utilizes readily available tools and small-caliber sheaths, making it easy for most vascular centers to adopt without requiring additional resources. Its straightforward design may help streamline workflows while enhancing patient safety.

## Introduction

Over the last years, endovascular aortic repair using branched or fenestrated stents-grafts (b- or f-EVAR) has evolved as a popular treatment for thoracoabdominal aortic aneurysm disease.^1^

Apart from the well-known four-vessel multibranched t-Branch® stent graft (Cook Medical Inc, Bloomington, Indiana), the E-nside^TM^ endograft (Artivion Inc, Kennesaw, Georgia) has also been introduced as a newer off-the-shelf device with four precannulated inner branches.^2,3^ The new device aims to reduce the time to cannulation (eg, in emergencies) or that of the intervention itself and, thus, of the radiation or contrast agent levels used.^3^ With these aims, several alternative custom-made devices with branches or combinations of branches, fenestrations, and scallops have been developed and introduced in clinical practice.^4^

In most instances, the available branches are cannulated by accessing a major artery in the upper extremity via open or percutaneous methods.^5^ This procedure is linked with several complications, including bleeding, dissection, stroke, and nerve injuries. To avoid such complications, the branches may well be cannulated via the generally safer femoral access by using steerable sheaths or even introducing a wire loop or a Snare® catheter in a second larger sheath.^6–8^ However, such a strategy comes with challenges as well. Specifically, considering that the mechanical robustness of a regular steerable sheath is not ideal, incidences of sheath dislocation when introducing the stent graft are not infrequent because of the increased rigidity of the stent graft in combination with the usual 180° angulation required during the intervention. Moreover, the wire loop-based solutions require a larger (≥ 12F) sheath, an option that might be associated with further complications (eg, arterial injuries) and subsequently extended operation times.^9–11^ Finally, custom-made solutions might require additional preparation time and lack standardization.^11,12^

Herein, we present our experience with a technique to avoid the upper extremity access while keeping the size of the femoral sheath at 7F and still allowing the tip of the sheath to be hooked into antegrade branches. Our approach further requires contralateral femoral access, yet with a minimum sheath size of 4F but uses commercially available and not custom-made configurations. The so-called “Lasso-Technique” is described in detail through four cases, as shown below. Furthermore, we provide insights into the advantages and disadvantages of the proposed approach while discussing further options for future improvements.

## Methods

### “Lasso-Technique” Description

**Figure 1.**
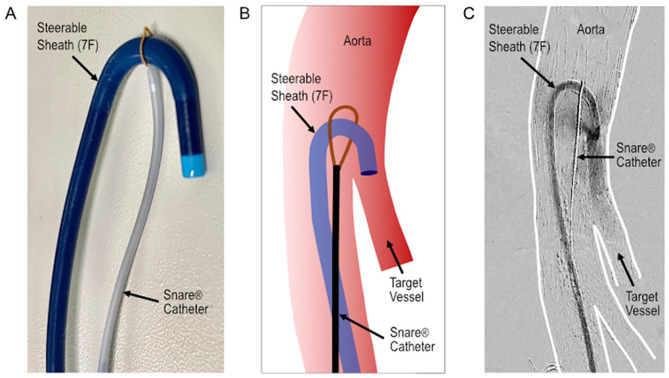
The “Lasso-Technique” with a steerable 7F sheath stabilized by a Snare® catheter. (A) Depiction of the required materials in vitro. (B) Schematic representation of the steerable 7F sheath placed in the target vessel orifice and stabilized by the Snare-catheter. (C) Intraoperative angiogram with required materials placed in vivo.

The “Lasso-Technique” (Figure 1) includes the following steps:

1. Bilateral retrograde puncture of the common femoral artery (CFA).
2. Insertion of a steerable sheath in 1 groin.
3. Insertion of a small-bore short sheath in the contralateral groin for the Snare® system.
4. Capture of the steerable sheath with the Snare® system after 90-degree deflection.
5. Stabilization of the Snare system with a mosquito clamp externally.
6. Stabilization of the steerable sheath by means of the Snare® system.
7. Conduction of the desirable endovascular procedure (eg, deployment of bridging stent, percutaneous mechanical thrombectomy, etc).

The technique was applied in a hybrid operation room (OR) setting that offers better flexibility and control.

**Figure 2.**
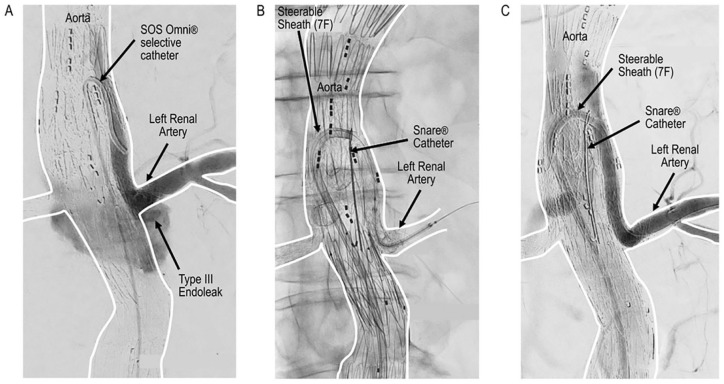
Case 1 presenting with type III endoleak from left renal branch after b-EVAR. (A) Type III endoleak caused by insufficient connection between bridging stents in left renal artery. (B) Transfemoral insertion of bridging stent using the “Lasso-Technique.” (C) Final angiogram showing the elimination of endoleak. b-EVAR: branched endovascular aortic repair.

**Figure 3.**
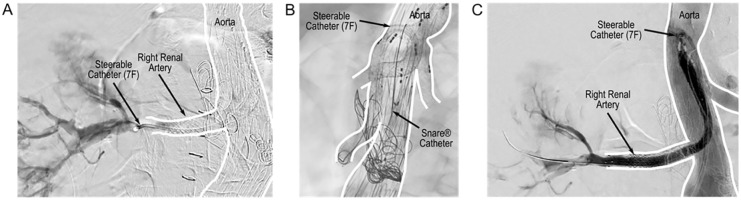
Case 2 presenting with occlusion of right renal artery branch. (A) Angiogram showing occlusion of right renal branch. (B) Cannulation of right renal branch using “Lasso-Technique.” (C) Final angiogram of right renal branch after rotational thrombectomy and lining with a stent graft.

**Figure 4.**
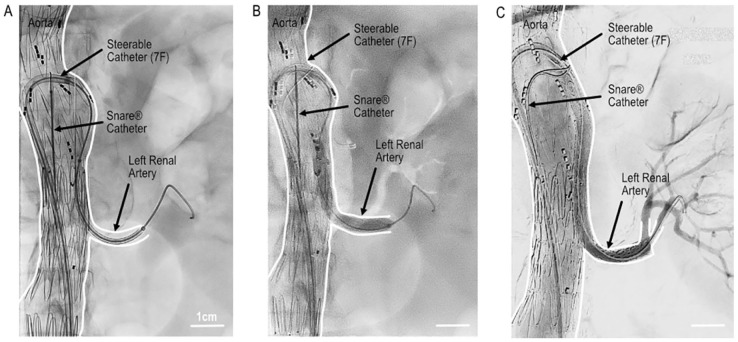
Case 3 demonstrating a thoracoabdominal aneurysm that was treated by transfemoral b-EVAR. (A) Insertion of a bridging stent in the left renal artery using the “Lasso-Technique.” (B) Implantation of the bridging stent. (C) Completion angiogram after incorporation of left renal artery. b-EVAR: branched endovascular aortic repair.

The described approach is further demonstrated via four (N=4) patient cases (case 1: male 81 years, case 2: male 73 years, case 3: female 75 years, and case four: male 85 years). In cases 1, 2, and 4, the patients were already treated with a branched prosthesis (t-Branch®, Cook Medical Inc). Furthermore, patients 1 and four were diagnosed (via computed tomography angiography, CTA) with a type III endoleak, while patient 2 was diagnosed with an occluded right renal branch/stent graft. In patient 3, a t-Branch® prosthesis was implanted via transfemoral access by applying our “Lasso-Technique” (Figures 1–4). The hybrid OR was equipped with a Philips Azurion® Flex-Arm System (Philips Healthcare, Inc, The Netherlands). All patients underwent general anesthesia and received systemic heparinization according to the usual guidelines.

During the interventions, we employed an Aptus TourGuide^TM^ Steerable Sheath (Aptus Endosystems Inc, Sunnyvale, California) (cases 1, 2, 3) and Oscor Destino Twist® (Oscor Inc, Palm Harbor, Florida), all in sizes 7F (case four), considering that the employed “Lasso-Technique” allows the use of any available steerable sheath.

After bilateral retrograde ultrasound-guided puncture of the CFA, a 5F or 4F-Terumo-10cm sheath was introduced in the one groin and a 7F steerable sheath in the contralateral one. The steerable sheath was positioned near the ostium to be cannulated. As a next step, a Snare® system (Multi-Snare 20 mm, Aimecs GmbH, Germany) was positioned in front of the steerable sheath. The steerable sheath was then angulated (90°) and captured by the Snare® sling. If possible, we tried to position the Snare® sling at the highest point of the angulated sheath. In parallel, the Snare® wire was fixed by a mosquito clamp at the external end of the Snare® catheter. In the cases presented, we utilized a 180° steerable sheath. In the presence of endografts with larger luminal diameters, there is a possibility that the open Snare® may not automatically slide over the angled sheath. To address this, it may be helpful to advance a wire through the steerable sheath into the loop of the snare. Thereafter, the snare can be retracted over the wire onto the tip of the sheath. Conversely, in situations involving smaller diameter grafts, where achieving even a 90° angle is not possible, stabilizing the sheath may pose a technical challenge.

Subsequently, the cannulation of the branch was done using an IM catheter (Cordis Inc, Santa Clara, California) and a 0.035” guidewire (Terumo BCT Inc., Japan), as usually. Under these circumstances, the angulation of the steerable sheath was increased to 180°. After successful cannulation and placing the wire in a distal to the cannulated vessel position, the 0.035” guidewire was changed to a Rosen guidewire of 260 cm length (Cook Medical Europe, Denmark), except in case 3, where we used the included dedicated Rotarex® 0.018” wire. In Figure 1, we provide a schematic representation of the principle of the employed “Lasso-Technique.”

### Ethical Approval and Informed Consent Statements

The local Ethics Committee waived the need for ethics approval and patient consent for the current study (study number: 2024-189-W-SB).

### Case Reports

#### Case 1

In case 1 (Figure 2), an 81-year-old male was diagnosed with an asymptomatic type III endoleak originating from the left renal artery and, specifically, from the connection between the renal branch and the left stent graft of the renal artery. The patient had received a successful treatment of a ruptured juxtarenal aneurysm (maximum diameter 67 mm) 4 days before the currently described intervention. The endoleak was again detected by means of aortography. The left renal branch was then cannulated, and a steerable 7F sheath was introduced through the left CFA. Unfortunately, the introduction of the selected stent graft (Gore Viabahn® VBX 6 × 59 mm, W.L. Gore Associates GmbH, Germany) led to repetitive sheath dislocations, so that, finally, the stent implantation turned out to be technically impossible. Moreover, a brachial access was performed 4 days ago, and we tried to avoid repetition to avoid possible complications. Thus, we decided to apply the “Lasso-Technique” and catch the tip of the steerable sheath with a Snare® catheter introduced via a second femoral access (4F sheath in the left CFA). By following this strategy, we re-cannulated the left renal branch and implanted the stent graft over a Rosen wire. Finally, after 2 balloon dilatations over the connection point between the renal branch and the left stent graft of the renal artery, the type III endoleak was successfully eliminated, as validated using angiography.

#### Case 2

In case 2 (Figure 3), a 73-year-old male with abdominal metastases of colon cancer (adenocarcinoma) was admitted with a subacute occlusion of the right renal artery after being treated by a t-Branch® prosthesis in another hospital 4 years ago. In a CTA 2 days ago, the branch was shown to be completely unobtrusive. The occlusion was detected in a second CTA (1 day before the intervention to rule out a pulmonary embolism by relevant symptoms). As a next step, we decided to perform an interventional recanalization of the right renal branch from the groin. The occluded right renal artery was primarily treated by Sofia® Plus® 6F aspiration catheter (Microvention Terumo, Aliso Viejo, California) with no satisfying angiographic results. After stabilizing the steerable sheath with the Snare® catheter, it was possible to introduce a 6F Rotarex® S (Straub Medical AG, Switzerland) system over the dedicated Rotarex® 0.018” wire (Figure 2). With the “Lasso-Technique,” we managed to perform both thrombectomy and atherectomy. Then, 2 stent grafts (Advanta® 6 × 100 mm [Getinge AB, Sweden] and Viabahn® VBX 6 × 40 mm [W.L. Gore Associates GmbH]) were delivered and expanded, enabling sufficient perfusion of the right renal artery. No increasing serum creatinine levels were observed in the following weeks after the intervention.

#### Case 3

In case 3 (Figure 4), a thoracoabdominal aortic aneurysm (maximum diameter 60 mm, 2× thoracic endograft, t-Branch®, distal aortic extension, 3 stent grafts) was completely treated via a transfemoral percutaneous access. After implantation of 2 thoracic endografts (Bolton Relay® Plus 30-30-209, Bolton Medical Inc, Sunrise, Florida and GORE® TAG® 31-31-200, W.L. Gore Associates GmbH), a t-Branch® prosthesis (Cook Medical Inc) along with a distal extension (22 mm) was implanted. In this case, all visceral and left renal branches were extended with stent grafts by using the “Lasso-Technique.” The left renal artery and the superior mesenteric artery were, respectively, supplied by a 6 × 59 mm Advanta® (Getinge AB, Sweden) stent graft; the coeliac trunk was supplied by a Viabahn® VBX 8 × 59 mm (W.L. Gore Associates GmbH). The right renal branch and the distal end of the t-Branch® prosthesis were not further recanalized to prevent spinal cord ischemia.

#### Case 4

**Figure 5.**
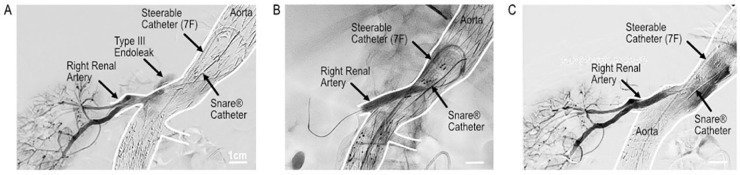
Case four with type III endoleak after b-EVAR. (A) Type III endoleak from the junction between the right renal branch and bridging stent. (B) Lining of right renal branch by implantation of a stent graft using the “Lasso-Technique.” (C) Final angiogram demonstrating no endoleak. b-EVAR: branched endovascular aortic repair.

In case four (Figure 5), an 85-year-old male was admitted with a type III endoleak after being treated several years ago by implantation of a thoracic endograft, an aortobiiliac endograft, and a custom-made device (3 branches and 1 fenestration for left renal artery; Cook Medical Inc) prosthesis. The transfemoral angiography showed an endoleak at the connection point between right renal branch and the implanted stent graft (Advanta® 6 × 38 mm, implanted in 2020). Thus, under these circumstances, the cannulation of the right renal branch was possible retrogradely. However, the introduction of the balloon (Mustang® 6 × 40 mm, Boston Scientific Inc, Marlborough, Massachusetts) was impossible because of its stiffness. The patient had undergone a transposition of the left subclavian artery (in 2018) and had 3 times right trans-brachial access, so we tried once more not to harm the right upper extremity by using the “Lasso-Technique.” We caught the 7F sheath with a Snare-catheter (via a second puncture on the left CFA and introduction of the 5F sheath) to stabilize the sheath. With this maneuver, we enabled the inflation of the balloon in order to bridge the connection with a 6 × 38 mm Advanta® stent graft (Getinge AB, Sweden). In the final angiography, no endoleak was identified.

## Discussion

The traditional way to cannulate antegrade branches in b-EVAR procedures requires upper extremity access. The latter, however, was shown to be associated with high radiation exposure,^13^ as well as other, most profound, complications. Thus, in order to avoid hematoma, nerve compression, and even stroke,^7^ interventionists usually perform the entire b-EVAR procedure via transfemoral access.^7,14^ Especially in small or calcified brachial arteries, the development of severe scarring after previous brachial artery cut down or transposition of the subclavian artery, the transfemoral access is even more desirable.

With the “Lasso-Technique,” heavily angulated branches are amenable to the transfemoral introduction of different balloon catheters, stent-graft implantation, or even treatment with mechanical rotational thrombectomy catheters.

Potential advantages compared to other relevant femoral access-based approaches, such as the previously described “preloaded wire technique,”^15^ are the: (i) fact that no wire impedes the tip of the steerable sheath to be hooked in branches, (ii) employment of smaller access/sheath diameters (eg, 7F and 4F vs 12F),^8–11^ and (iii) use of frequently used and not custom-made materials,^11,12^ and, thus, the possible achievement of faster times considering that no construction time is required (even if further studies are still needed to prove it).

Of course, as with all other techniques, the employed approach used herein comes with possible limitations. More specifically, a limitation of this maneuver is the resulting torqued anatomy/configuration, but, as presented in all our cases, this limitation turned out to be a minor technical challenge. Possibly, the “Lasso-Technique” might perform worse in tortuous aortic anatomies. However, this remains to be investigated in future targeted studies. Furthermore, in cases where the Snare-catheter might not be correctly placed at the apex of the steerable sheath, the Snare® catheter could slip off distally or proximally. Nevertheless, in such cases, it is technically feasible to replace the Snare® catheter very quickly.

Finally, based on our experience, one of the disadvantages of the employed method is that it requires vascular access from both groins, which can increase the risk of complications such as arterial injury, hemorrhage, or occlusion compared to other techniques (eg, the preloaded wire technique). However, performing an ultrasound-guided puncture can help minimize the rates of these complications.

## Conclusion

The “Lasso-Technique” allows for introducing balloon catheters, stents or stent grafts, or mechanical rotational catheters (eg, Rotarex®, Straub Medical AG, Switzerland) into antegrade branches during b-EVAR procedures. The technique was proven reproducible in a small case series. Avoiding the brachial access may result in reduced radiation exposure and lower patient morbidity associated with relevant complications and technical challenges. Furthermore, the presented technique allows the tip of the sheath to be hooked into antegrade branches employing small-sized sheaths. Finally, the technique is simple and requires readily available tools found in any specialized health care unit. Future observational studies should document the operational time and radiation exposure of the proposed technique in comparison to other methods. Such relevant studies will further support the endorsement of the technique.
